# Psychological burden as the primary determinant of suicidal ideation in patients with temporomandibular disorders

**DOI:** 10.22514/jofph.2026.051

**Published:** 2026-07-12

**Authors:** Tatiana Sella Tunis, Itzhak Agayev, Aya Pessing-Shabi, Yifat Manor, Waseem Abboud, Shoshana Reiter

**Affiliations:** ^1^Department of Orthodontics, The Maurice and Gabriela Goldschleger School of Dental Medicine, Gray Faculty of Medical and Health Sciences, Tel Aviv University, 6997801 Tel Aviv, Israel; ^2^The Maurice and Gabriela Goldschleger School of Dental Medicine, The Faculty of Medical and Health Sciences, Gray Faculty of Medical and Health Sciences, Tel Aviv University, 6997801 Tel Aviv, Israel; ^3^Department of Oral Pathology, Oral Medicine, and Maxillofacial Imaging, The Maurice and Gabriela Goldschleger School of Dental Medicine, Gray Faculty of Medical and Health Sciences, Tel Aviv University, 6997801 Tel Aviv, Israel; ^4^Department of Oral and Maxillofacial Surgery, Kaplan Medical Center, 7610001 Rehovot, Israel; ^5^Department of Oral and Maxillofacial Surgery, The Maurice and Gabriela Goldschleger School of Dental Medicine, Gray Faculty of Medical and Health Sciences, Tel Aviv University, 6997801 Tel Aviv, Israel; ^6^Unit of Oral and Maxillofacial Surgery, Bnei Zion Medical Center, 3339419 Haifa, Israel; ^7^Department of Neurology, Institute of Movement Disorders, Sheba Medical Center, 5262100 Ramat Gan, Israel

**Keywords:** Chronic pain, IASP, Psychological distress, Suicidal ideation, Temporomandibular disorders

## Abstract

**Background**: Assessing suicidal ideation (SI) in patients with primary temporomandibular disorders (TMD) is crucial for its early identification and intervention. This study aimed to investigate the biopsychosocial factors associated with SI in the overall TMD sample and across subgroups of painful TMD patients. **Methods**: A total of 441 TMD patients were enrolled. TMD was diagnosed using the diagnostic criteria for TMD, and SI was assessed via item 9 of the Patient Health Questionnaire-9. SI prevalence was compared across painful TMD subgroups, stratified by pain duration, and classified according to the International Association for the Study of Pain (IASP) diagnostic criteria. Sociodemographic, Axis I and II, and pain characteristics were compared between the SI and non-SI groups using the Chi-square and Mann-Whitney tests. Binary logistic regression, incorporating pain persistence and depression, was performed to identify the key factors independently associated with SI. **Results**: In the overall TMD sample, encompassing both painful and non-painful patients, 8.2% reported SI. Among the painful TMD subgroups, SI prevalence was 5.7% in acute cases and 12.0% in chronic cases by pain duration, rising to 20.9% when chronic pain was defined by IASP criteria (*p* < 0.001). Patients with SI were more frequently divorced, had higher rates of TMD-attributed headache and myofascial pain with referral, and reported greater perceived pain intensity. They also exhibited markedly elevated depression, anxiety, non-specific physical symptoms, pain catastrophizing, and overall distress (*p* < 0.001), as well as greater disability (*p* = 0.004). Pain duration was not independently associated with SI, whereas depression emerged as a significant independent predictor (*p* < 0.001). **Conclusions**: These findings highlight the substantial psychological burden in TMD patients, particularly those with chronic pain, and underscore the critical role of depression in SI. Incorporating routine biopsychosocial assessment and SI screening into TMD clinical practice is, therefore, highly recommended.

## 1. Introduction

According to the World Health Organization (WHO), over 720,000 individuals die by suicide annually, making it the third leading cause of death among individuals aged 15–29 [1]. From 2015–2019, the estimated prevalence of suicidal ideation (SI) in the general adult population in the United States ranged from about 4 to 6% [2, 3]. Similar SI rates (5.5%) were reported in Israel [4]. Early identification of individuals experiencing SI is critical, since more than 60% of suicide attempts occur within the year following SI [5]. Transition rates from SI to suicide attempts were reported to range from 2.6 to 37%; mental health disorders, particularly depression and anxiety, were associated with an increased risk of attempting suicide [6].

Chronic pain has been consistently identified as a significant risk factor for suicidality, regardless of its underlying etiology [7]. Moreover, chronic pain-related factors such as mental health [8], sleep disturbances [9], comorbid chronic pain conditions [10], multi-health conditions [11], and physical pain [12] were also associated with a higher likelihood of suicidal thoughts or destructive behavior. SI has been extensively examined across chronic pain conditions; higher rates of suicidality have been reported among individuals with fibromyalgia [13], back pain, migraine, non-migraine headache, neck pain, and arthritis [14]. In line with this, the WHO identified chronic pain as a potential risk factor for suicide, along with other established risk factors, such as prior suicide attempts, mental health disorders, substance use, financial hardship, and a family history of suicide [1].

Temporomandibular disorders (TMD) constitute a heterogeneous group of musculoskeletal and neuromuscular conditions affecting the temporomandibular joint (TMJ) complex, as well as the associated musculature and osseous structures [15]. The etiology of TMD is multifactorial, involving biological, environmental, social, emotional, and cognitive components, consistent with the biopsychosocial model [16]. In a recent systematic review and meta-analysis, the global prevalence of TMD was estimated at 35.4%, based on studies that used the diagnostic criteria for TMD (DC/TMD). Therefore, it was concluded that TMD may represent significant medical, financial, and social burdens [17]. Clinical manifestations of TMD range from mild, self-limiting discomfort to chronic pain and dysfunction, resulting in significant disability and a diminished quality of life. Accumulating evidence suggests that roughly one-third to one-half of patients with TMD develop a chronic condition over time [18]. It has been estimated that about 85% of the costs associated with TMD treatment are largely attributable to a relatively small proportion of patients who develop chronic TMD [19]. Consequently, a key clinical challenge is to identify early those patients with established or potential chronic pain, as well as those patients expressing SI and require individualized treatment strategies and considerations to optimize their outcomes [20].

Currently, the DC/TMD clinical examination protocol is considered the most widely used protocol for diagnosing TMD in both clinical and research settings; notably, it has high validity and reliability [21]. In accordance with the biopsychosocial model, the DC/TMD protocol provides a comprehensive assessment that includes not only physical findings evaluated through the Axis I protocol, but also a psychosocial evaluation (Axis II). More specifically, it incorporates validated questionnaires to screen for depression, generalized anxiety, non-specific physical symptoms, and pain-related disability. Regarding SI, item 9 of the Patient Health Questionnaire-9 (PHQ-9), used to assess depressive symptoms, queries about SI during the preceding two weeks and may facilitate early identification of patients at elevated risk for suicide. Consequently, all information needed to classify TMD patients as having chronic painful TMD as well as information regarding those patients reporting SI is available to the treating clinician when applying the DC/TMD protocol. However, the complexity of using the entire DC/TMD protocol in non-academic settings [22] has led many clinicians to adopt the brief DC/TMD (bDC/TMD) protocol for non-specialist practice [23]. However, this abbreviated approach excludes the evaluation of SI.

In line with the biopsychosocial model represented by the DC/TMD protocol, the International Association for the Study of Pain (IASP) incorporated this framework into its updated definition of chronic primary pain. It defined it as “pain in one or more anatomical regions that persists or recurs for longer than three months and is associated with significant emotional distress (*e.g.*, anxiety, anger, frustration, or depressed mood) and/or significant functional disability (interference with activities of daily life and participation in social roles), and is not better accounted for by another diagnosis” [24]. Accordingly, the IASP classifies chronic TMD pain as a chronic primary pain within the category of chronic primary headache or orofacial pain. However, in the same article [24], the specific description of chronic primary TMD pain provided by Nicholas *et al*. [24] largely relies on temporal characteristics, defining chronic primary TMD pain as “chronic orofacial pain that occurs for at least 2 hours per day on at least 50% of the days over at least 3 months”. Consequently, this definition may encourage reliance on pain duration as the primary indicator of chronicity in TMD, potentially overlooking other relevant psychosocial factors.

The present study aimed to investigate the socio-demographic, clinical (Axis I), psychosocial (Axis II), and pain-related factors associated with SI in patients with primary TMD. We also examined whether SI prevalence varies across distinct psychological stress periods and among different painful TMD subtypes. We hypothesized that certain TMD subgroups would show higher SI prevalence and that specific clinical and psychosocial factors would be significantly associated with SI. Better understanding of these associations is clinically important, since it may help identify patients at elevated risk for SI. It may also guide and evaluate targeted interventions addressing both TMD-related signs and symptoms as well as psychosocial burden using a multidisciplinary approach.

## 2. Materials and methods

### 2.1 Study sample

This retrospective study screened all (n = 1240) consecutive patients who were examined at the Orofacial Pain Clinic at Tel Aviv University, Israel, from January 2015–October 2025.

Inclusion criteria were as follows: (1) a confirmed diagnosis of primary TMD according to the DC/TMD protocol, (2) age ≥18 years, and (3) completion of the DC/TMD questionnaire, including data required for evaluating chronic pain according to the IASP.

A total of 799 patients were excluded based on the following exclusion criteria: age <18 years, a missing DC/TMD questionnaire, missing SI information, non-TMD patients according to the DC/TMD (not fulfilling DC/TMD criteria for TMD), secondary TMD conditions (including immunological (*e.g.*, rheumatoid arthritis), inflammatory (*e.g.*, diffuse sclerosing osteomyelitis), neuromuscular disorders (*e.g.*, dystonia)), trauma-related conditions and structural conditions (*e.g.*, coronoid hyperplasia), tumor-related conditions (*e.g.*, trismus secondary to radiation), bruxism without TMD, obstructive sleep apnea, and non-TMD orofacial pain conditions (*e.g.*, odontogenic pain, neuropathic pain, primary headache disorders, cervical-related pain, occlusal dysesthesia, burning mouth syndrome (BMS), and tumors).

The final population included in the study consisted of 441 patients with primary TMD. This study was approved by the Tel Aviv University Institutional Ethical Committee (ID: 0004950-3). All patients signed a form agreeing that their data could be used anonymously for research purposes.

### 2.2 Clinical assessment and diagnostic tools

The 10-year study period (2015–2025) was divided into three intervals based on different psychological impacts on SI: pre-Coronavirus Disease (COVID) (January 2015–February 2020), COVID (March 2020–September 2023), and the war period in Israel (October 2023–October 2025).

All patients were examined by senior staff members certified in DC/TMD training and the calibration course given at the Department of Orofacial Pain and Jaw Function, Faculty of Odontology, Malmö University, Sweden. The official Hebrew version of the DC/TMD protocol was used to collect both Axis I and Axis II data for diagnosing TMD [25].

Physical diagnoses: Axis I diagnoses of the DC/TMD were used to identify and classify physical TMD diagnoses, which were categorized as non-painful (intra-articular disorders (IAD), degenerative joint disease (DJD), and subluxation) or painful (local myalgia, myofascial pain with referral, arthralgia, and TMD-attributed headache).

Psychosocial assessment: It was conducted using the Axis II component of the DC/TMD criteria, employing the Patient Health Questionnaire-4 (PHQ-4), an ultra-brief, validated tool for screening anxiety and depression in clinical and non-clinical populations [26, 27]. PHQ-4 comprised four items: the first two (GAD-2) assessed generalized anxiety, and the last two (PHQ-2) assessed depressive symptoms. A score ≥3 on either subscale indicated a positive screen for anxiety or depression, respectively. Reported diagnostic accuracy includes a sensitivity of 83% and a specificity of 90% for major depressive disorder (PHQ-2) and a sensitivity of 88% for generalized anxiety disorder (GAD-2) [28]. Anxiety (items 1–2) and depression (items 3–4) subscale scores were calculated separately. Overall psychological distress was assessed using the total PHQ-4 score (range 0–12) and categorized as none/low (0–5) or moderate/severe (6–12) [29]. A total PHQ-4 score of 6 or higher (moderate to severe distress) was recommended as an indicator that additional clinical evaluation, targeted screening, or referral to mental health services may be warranted [26]. Additional Axis II measures included PHQ-15 to assess non-specific physical symptoms and the Graded Chronic Pain Scale (GCPS) (version 2.0) to evaluate pain-related disability, categorized as either low (grades 0–2) or high (grades 3–4).

Pain persistence: It was assessed using the DC/TMD item regarding the number of days with facial pain in the previous 6 months and was classified as none (0 days), low (<90 days), or high (≥90 days). Further psychosocial assessment included the Pain Catastrophizing Scale (PCS) [30] (the Hebrew-validated version) [31].

SI assessment: It was assessed using item 9 of PHQ-9, which is included in the DC/TMD Axis II protocol: “Over the past 2 weeks, how often have you been bothered by the following problem: thinking that you would be better off dead or of hurting yourself in some way?”. Response options included “not at all”, “several days”, “more than half the days”, “nearly every day”. Patients were classified as “non-SI” if they responded “not at all” to the assessment question. All other responses resulted in classification as “SI”.

Within a multidisciplinary framework, all Axis II data, including PHQ-9 item 9, were systematically reviewed and scored by the examining clinician at the initial visit following completion of the DC/TMD questionnaire. Suicide risk was further evaluated by the attending physician based on prior psychiatric diagnoses, current psychotropic medication use, ongoing mental health treatment, and inquiring about the presence of suicidal intent or plan. Based on this evaluation, patients were referred to mental health services for further assessment and management. Concurrently, TMD-related pain and dysfunction were addressed within our clinic, with coordination between dental and mental health providers to ensure integrated and comprehensive care. Patients were reassessed accordingly during follow-up visits.

Acute/chronic painful TMD definitions: Patients diagnosed with at least one painful Axis I condition were classified as having acute or chronic painful TMD based on two definitions:

1. According to pain duration only: acute painful TMD was defined as pain persisting for <90 days during the previous six months, whereas chronic painful TMD was defined as pain persisting for ≥90 days.

2. According to the IASP general definition of chronic primary pain [24]: Although a detailed protocol for implementing the DC/TMD data to define chronic pain according to the IASP was not available, we defined chronic primary pain following the IASP definition: pain persisting or recurring for more than three months and associated with either significant emotional distress and/or substantial functional disability, not better accounted for by another diagnosis. Emotional distress was measured using the PHQ-4 total score (range 0–12), with scores ≥6 indicating moderate to severe distress, consistent with recommendations for further clinical evaluation or referral to mental health services [26, 29]. Functional disability was assessed using the GCPS instrument; grades 3 and 4 represented high disability and interference with daily activities and social participation [32]. Patients with a pain persistence score of ≥90 days and who met either criterion, *i.e.*, moderate/severe distress and/or high GCPS disability, were classified as having chronic primary pain according to the IASP framework. This approach was considered the most practical way to align the available DC/TMD data with the IASP framework.

### 2.3 Statistical analysis

The sample size was based on all available cases that met the inclusion criteria and were selected from patients who received treatment between 2015 and 2025.

The data were analyzed using IBM SPSS Statistics for Windows, version 29.0.2 (IBM Corp., Armonk, NY, USA). Categorical variables were described as frequencies and percentages. The distribution of continuous variables was evaluated using histograms and assessed using the Kolmogorov-Smirnov test. Variables were presented as median and interquartile range (IQR) due to the non-normal distribution. Chi-square and Fisher’s Exact tests were used to examine associations between SI groups and categorical variables. Additionally, the Chi-square test was used to examine differences in SI prevalence between study periods. Continuous and ordinal variables were compared between SI groups using the Mann-Whitney U test. Effect sizes for differences between the SI and non-SI groups were calculated using Cohen’s *d* for continuous variables and Cramer’s *V* for categorical variables. Binary logistic regression analysis (the enter method) was performed to determine how pain persistence and the depression level (independent variables) contributed to the likelihood of SI (dependent variable). A *p*-value < 0.05 was considered statistically significant for all analyses.

## 3. Results

### 3.1 Study sample

The study population included 441 primary TMD patients, comprising 131 males (29.7%) and 310 females (70.3%). The mean age was 36.64 ± 14.48 years, and the median age was 32 years (IQR: 25.25–45). Tables 1,2,3,4 summarize the demographic and socioeconomic parameters, pain characteristics, as well as the Axis I diagnoses and Axis II screening evaluations of the sample.

**Table 1.** Socioeconomic and demographic characteristics, by SI status.

Socio-economic/demographic parameters		Non-SI TMD (n = 405)	SI TMD (n = 36)	Total TMD (n = 441)	*p*-value*	Effect size
Gender, n (%)						
	Male	122 (30.1%)	9 (25.0%)	131 (29.7%)	0.519	0.031
	Female	283 (69.9%)	27 (75.0%)	310 (70.3%)		
Age (yr)						
	Mean ± SD	36.26 ± 14.30	40.89 ± 16.05	36.64 ± 14.48	0.094	−0.320
	Median (IQR)	32 (25–46)	39 (26–53.25)	32 (25.25–45)		
Education:						
	Elementary School	9/399 (2.3%)	0/31 (0.0%)	9/430 (2.1%)	0.489	0.061
	High School Student	122/399 (30.6%)	12/31 (38.7%)	134/430 (31.2%)		
	College Student	63/399 (15.8%)	5/31 (16.1%)	68/430 (15.8%)		
	College Graduate	108/399 (27.1%)	8/31 (25.8%)	116/430 (27.0%)		
	Professional or Postgraduate Level	97/399 (24.3%)	6/31 (19.4%)	103/430 (24.0%)		
	Valid Responses	399/405 (98.5%)	31/36 (86.1%)	430/441 (97.5%)		
Income:						
	Very Low	16/378 (4.2%)	3/30 (10.0%)	19/408 (4.7%)	0.254	0.126
	Low	31/378 (8.2%)	2/30 (6.7%)	33/408 (8.1%)		
	Average	231/378 (61.1%)	20/30 (66.7%)	251/408 (61.5%)		
	High	91/378 (24.1%)	3/30 (10.0%)	94/408 (23.0%)		
	Very High	9/378 (2.4%)	2/30 (6.7%)	11/408 (2.7%)		
	Valid Responses	378/405 (93.3%)	30/36 (83.3%)	408/441 (92.5%)		
Marital Status:						
	Married/Living as Married	197/402 (49.0%)	12/33 (36.4%)	209/435 (48.0%)	**0.046**	0.138
	Divorced/Separated	17/402 (4.2%)	5/33 (15.2%)	22/435 (5.1%)		
	Widowed	9/402 (2.2%)	1/33 (3.0%)	10/435 (2.3%)		
	Never Married	179/402 (44.5%)	15/33 (45.5%)	194/435 (44.6%)		
	Valid Responses	402/405 (99.3%)	33/36 (91.6%)	435/441 (98.6%)		

SI: Suicide Ideation; TMD: Temporomandibular Disorder; SD: Standard Deviation; IQR: Interquartile Range. Values are presented as n/N (%), where n = the number of participants with the characteristic and N = the number of valid responses for that variable. *p-values (non-responders excluded from the test) for the differences between the SI and non-SI groups. Significant p-values are shown in bold (p < 0.05).

**Table 2.** Axis I TMD diagnosis rates, by SI status.

Axis I Diagnoses		Non-SI TMD (n = 405)	SI TMD (n = 36)	Total TMD (n = 441)	*p*-value*	Effect size
Painful category						
	Local Myalgia	181 (44.7%)	10 (27.8%)	191 (43.3%)	0.050	0.093
	Myofascial Pain with Referral	138 (34.1%)	21 (58.3%)	159 (36.1%)	**0.004**	0.138
	Arthralgia	104 (25.7%)	7 (19.4%)	111 (25.2%)	0.409	0.039
	Headache Attributed to TMD	155 (38.3%)	21 (58.3%)	176 (39.9%)	**0.018**	0.112
Non-painful category						
	DJD	82 (20.2%)	7 (19.4%)	89 (20.2%)	0.908	0.005
	IAD	184 (45.4%)	11 (30.6%)	195 (44.2%)	0.085	0.082
	Subluxation	55 (13.6%)	5 (13.9%)	60 (13.6%)	0.999	0.002

SI: Suicide Ideation; TMD: Temporomandibular Disorder; DJD: Degenerative Joint Disease; IAD: Intra-Articular Disorders. *p-values for the differences between the SI and non-SI groups. Significant p-values are shown in bold (p < 0.05).

**Table 3.** Pain-related variables, by SI status.

Pain characteristics		Non-SI TMD (n = 405)	SI TMD (n = 36)	Total TMD (n = 441)	*p*-value*	Effect size
Jaw/Temple/Ear Pain in the Last 30 Days						
	No pain	33/360 (9.2%)	2/32 (6.2%)	35/392 (8.9%)	0.750	0.038
	Intermittent Pain	222/360 (61.7%)	19/32 (59.4%)	241/392 (61.5%)		
	Constant Pain	105/360 (29.2%)	11/32 (34.4%)	116/392 (29.6%)		
	Valid Responses	360/405 (88.9%)	32/36 (88.9%)	392/441 (88.9%)		
Temple Headaches in the Last 30 Days						
	No	182/394 (46.2%)	10/36 (27.8%)	192/430 (44.7%)	**0.033**	0.103
	Yes	212/394 (53.8%)	26/36 (72.2%)	238/430 (55.3%)		
	Valid Responses	394/405 (97.3%)	36/36 (100.0%)	430/441 (97.5%)		
CPI						
	Mean ± SD	48.10 ± 27.3	64.0 ± 28.0	49.38 ± 27.66	**<0.001**	−0.581
	Median (IQR)	53.3 (30–67.5)	73.3 (50–83.3)	53.33 (30–70)		
	Valid Responses	402/405 (99.3%)	35/36 (97.2%)	437/441 (99.1%)		
Pain onset						
	<1 mon	4/357 (1.1%)	0/33 (0.0%)	4/389 (1.0%)	0.809	0.037
	1–6 mon	88/357 (24.6%)	9/33 (27.3%)	97/389 (24.9%)		
	7–11 mon	21/357 (5.9%)	2/33 (6.1%)	23/389 (5.9%)		
	1–2 yr	70/357 (19.6%)	6/33 (18.2%)	76/389 (19.5%)		
	>2 yr	174/357 (48.7%)	15/33 (45.5%)	189/389 (48.6%)		
	Valid Responses	357/405 (88.1%)	33/36 (91.7%)	389/441 (88.2%)		
Pain Persistence Score: the number of pain days in the last 6 months						
	No pain	40/405 (9.9%)	3/36 (8.3%)	43/441 (9.8%)	0.060	0.113
	<90 d	199/405 (49.1%)	11/36 (30.6%)	210/441 (47.6%)		
	≥90 d	166/405 (41.0%)	22/36 (61.1%)	188/441 (42.6%)		
	Valid Responses	405/405 (100.0%)	36/36 (100.0%)	441/441 (100.0%)		

SI: Suicide Ideation; TMD: Temporomandibular Disorder; CPI: Characteristic Pain Intensity; SD: Standard Deviation; IQR: Interquartile Range. Values are presented as n/N (%), where n = the number of participants with the characteristic and N = the number of valid responses for that variable. *p-values (non-responders were excluded from the test) regarding the differences between the SI and non-SI groups. Significant p-values are shown in bold (p < 0.05).

**Table 4.** Axis II TMD evaluations, by SI status.

Axis II evaluation: Categories/Values		Non-SI TMD (n = 405)	SI TMD (n = 36)	Total TMD (n = 441)	*p*-value*	Effect size
Depression Level						
	Normal	335/403 (83.1%)	7/36 (19.4%)	342/439 (77.9%)	**<0.001**	0.421
	Probable depression	68/403 (16.9%)	29/36 (80.6%)	97/439 (22.1%)		
	Valid Responses	403/405 (99.5%)	36/36 (100.0%)	439/441 (99.5%)		
Anxiety Level						
	Normal	295/405 (72.8%)	8/36 (22.2%)	303/439 (69.0%)	**<0.001**	0.299
	Probable anxiety	110/405 (27.2%)	28/36 (77.8%)	138/439 (31.4%)		
	Valid Responses	405/405 (100.0%)	36/36 (100.0%)	439/441 (99.5%)		
Distress Level (PHQ-4)						
	Normal	224/405 (55.3%)	None	224/441 (50.8%)	**<0.001**	0.418
	Mild	103/405 (25.4%)	7/36 (19.4%)	110/441 (24.9%)		
	Moderate	42/405 (10.4%)	11/36 (30.6%)	52/441 (11.8%)		
	Severe	36/405 (8.9%)	18/36 (50.0%)	54/441 (12.2%)		
	Valid Responses	405/405 (100.0%)	36/36 (100.0%)	441/441 (100.0%)		
Non-specific Physical Symptoms (PHQ-15)						
	Normal	192/403 (47.6%)	7/36 (19.4%)	199/439 (45.3%)	**<0.001**	0.266
	Mild	124/403 (30.8%)	10/36 (27.8%)	134/439 (30.5%)		
	Moderate	66/403 (16.4%)	9/36 (25.0%)	75/439 (17.1%)		
	Severe	21/403 (5.2%)	10/36 (27.8%)	31/439 (7.1%)		
	Valid Responses	403/405 (99.5%)	36/36 (100.0%)	439/441 (99.5%)		
Graded Chronic Pain Scale (GCPS) (0–IV)						
	0: None	49/395 (12.4%)	3/34 (8.8%)	52/429 (12.1%)	**0.004**	0.158
	I: Low Intensity (No Disability)	113/395 (28.6%)	3/34 (8.8%)	116/429 (27.0%)		
	II: High Intensity (No Disability)	153/395 (38.7%)	15/34 (44.1%)	168/429 (39.2%)		
	III: Moderately Limiting	40/395 (10.1%)	5/34 (14.7%)	45/429 (10.5%)		
	IV: Severely Limiting	40/395 (10.1%)	8/34 (23.5%)	48/429 (11.2%)		
	Valid Responses	395/405 (97.5%)	34/36 (94.4%)	429/441 (97.3%)		
Pain-Related Disability						
	No Disability	318/398 (79.9%)	21/34 (61.8%)	339/432 (78.5%)	**0.014**	0.119
	Moderate/severe disability	80/398 (20.1%)	13/34 (38.2%)	93/432 (21.5%)		
	Valid Responses	398/405 (98.3%)	34/36 (94.4%)	432/441 (98.0%)		
Pain Catastrophizing Scale (PCS)						
	Low	256/403 (63.5%)	9/36 (25.0%)	265/439 (60.4%)	**<0.001**	0.297
	Intermediate	65/403 (16.1%)	3/36 (8.3%)	68/439 (15.5%)		
	High	82/403 (20.3%)	24/36 (66.7%)	106/439 (24.1%)		
	Valid Responses	403/405 (99.5%)	36/36 (100.0%)	439/441 (99.5%)		

SI: Suicide Ideation; TMD: Temporomandibular Disorder; PHQ: Patient Health Questionnaire. Values are presented as n/N (%), where n = the number of participants with the characteristic and N = the number of valid responses for that variable. *p-values (non-responders were excluded from the test) regarding the differences between the SI and non-SI groups. Significant p-values are shown in bold (p < 0.05).

### 3.2 SI prevalence in TMD patients

In the sample, 8.2% (n = 36) reported SI and were classified as in the SI group. The rest of the sample (n = 405; 91.84%) stated that they had no thoughts regarding SI and were classified into the non-SI group (Fig. 1A). Among those individuals who had reported SI, two-thirds (n = 23; 63.9%) had experienced SI for several days, n = 6 (16.7%) for more than half the days, whereas n = 7 (19.4%) had experienced SI nearly every day.

### 3.3 SI prevalence by study periods

The total number of patients was 218 in the pre-COVID period and 130 and 93 in the COVID and war periods, respectively. The prevalence of SI was 10.1% (n = 22), 6.2% (n = 8), and 6.5% (n = 6) across the pre-COVID, COVID, and war periods, respectively, with no significant differences among periods (*p* = 0.358, Cramer’s *V* = 0.07). The number of SI events within each study period was relatively small, thus limiting the statistical power to detect differences between groups. Therefore, the absence of significant differences across periods should be interpreted cautiously and considered exploratory.

### 3.4 SI prevalence by painful TMD groups

Among patients with painful TMD, a significant difference in SI prevalence was observed between the acute and chronic primary pain groups (Fig. 1B,C). Patients with chronic painful TMD, defined according to pain duration only, reported nearly twice the prevalence of SI compared with those with acute painful TMD (12% *vs.* 5.7%, respectively; *p* = 0.037). When chronic pain was defined according to the IASP criteria, the prevalence of SI increased to 20.9%, a 3.6-fold higher rate than in the acute pain group (Fig. 1B,D; *p* < 0.001). Furthermore, a comparison between patients who met the IASP diagnostic criteria for chronic pain (n = 91, Fig. 1D) and those who reported high pain persistence (≥90 pain days in the preceding 6 months) but did not meet the IASP criteria due to low distress and disability levels (n = 84, Fig. 1E) revealed a marked difference in their SI prevalence. Only two patients (2.4%) in the latter group reported SI, compared with 20.9% of patients meeting the IASP chronic pain criteria, reflecting an 8.7-fold difference (*p* < 0.001) (Fig. 1D,E).

**Fig. 1.** Prevalence of suicidal ideation. (A) The total TMD sample, (B) acute painful TMD, (C) chronic painful TMD, (D) chronic painful TMD meeting the IASP criteria, and (E) chronic painful TMD excluded by the IASP criteria. Red denotes patients with SI, and blue denotes patients without SI. SI: suicidal ideation; TMD: temporomandibular disorder; PPS: pain persistence score; IASP: International Association for the Study of Pain.

### 3.5 Comparison between TMD patients with and without SI

#### 3.5.1 Demographics and socioeconomic characteristics

Significant differences were observed in marital status, *e.g.*, TMD patients with SI had a significantly higher prevalence of divorce and were considerably less likely to be married or living with a spouse (*p* = 0.046). No significant differences were found with respect to gender, age, education level, and income (*p* > 0.094) (Table 1).

#### 3.5.2 Axis I diagnoses

The prevalence of myofascial pain with referral and headache attributed to TMD was significantly higher among TMD patients with SI compared with TMD patients without SI (*p* < 0.018). The prevalence of other painful and non-painful diagnoses was similar between the groups (Table 2). Overall, 84% of patients without SI and 91.7% of patients with SI had at least one painful diagnosis, whereas 16% and 8.3%, respectively, had at least one non-painful diagnosis; there was no significant difference between the SI and non-SI groups (*p* = 0.219).

#### 3.5.3 Pain characteristics

Individuals with SI reported a significantly higher pain intensity and a greater prevalence of temporal headaches in the last 30 days compared with the non-SI TMD group (*p* = 0.033) (Table 3). Pain persistence also differed between the groups: 61.1% of patients with SI reported pain for 90 days or more in the last 6 months, compared with 41% of non-SI patients; however, this difference was not statistically significant (*p* = 0.060). No significant differences in pain onset were observed between the groups (*p* = 0.809) (Table 3). Patients with SI reported higher pain levels, as reflected by their characteristic pain intensity (CPI) score (*p* < 0.001).

#### 3.5.4 Axis II evaluation

Individuals with SI exhibited significantly higher levels of psychological distress and pain-related psychosocial burden across multiple Axis II evaluation measures. Compared with those without SI, they exhibited markedly higher rates of probable depression, anxiety, non-specific physical symptoms, pain catastrophizing, and overall distress (*p* < 0.001) (Table 4). Notably, individuals with SI also exhibited a higher prevalence of moderate/severe disability, as assessed by their GCPS level (*p* = 0.004), as well as greater pain-related disability (*p* = 0.014) (Table 4).

#### 3.5.5 Logistic regression analysis

Depression level was the only variable significantly associated with SI (*p* < 0.001) (Table 5). The overall model was statistically significant (*p* < 0.001) with a Nagelkerke *R*^2^ value of 0.308 and a Cox & Snell *R*^2^ value of 0.133, along with the results of the Hosmer-Lemeshow goodness-of-fit test (*p* = 0.727), and Variance Inflation Factor (VIF) <5.

**Table 5.** Logistic regression model summary.

Variable	Exp (B)	Lower CI	Upper CI	*p*-value*
Pain Persistence Score <90 d *vs.* no pain	0.90	0.21	3.83	0.890
Pain Persistence Score ≥90 d *vs.* no pain	1.16	0.29	4.59	0.836
Depression Level	19.33	8.00	46.71	**<0.001**

*Significant p-values are shown in bold (p < 0.05). CI: Confidence Interval; Exp (B): Exponentiated B.

## 4. Discussion

This study examined the prevalence of suicidal ideation (SI) and its associated factors among patients with primary temporomandibular disorders (TMD), using different criteria for chronic painful conditions.

TMD patients reporting SI differed from patients without SI across multiple domains of the biopsychosocial model. When the demographic and socioeconomic factors were examined, individuals with SI were more frequently divorced and less often married or living as married, supporting an association between reduced social support or social isolation and higher SI rates [33].

Regarding the pain characteristics, patients with SI reported higher pain intensity, consistent with previous findings linking greater pain severity to increased suicidal vulnerability [34]. However, pain onset did not differ significantly between patients with and without SI.

Regarding the Axis I and II findings, patients reporting SI showed higher rates primarily of myofascial pain with referral and headache attributed to TMD. Specifically, temple headache was reported by 73% of patients in the SI group, compared with 52% in the non-SI group, highlighting the importance of assessing SI in patients presenting with prominent headache symptoms [35]. In contrast to the relatively limited contribution of most Axis I diagnoses, Axis II measures showed strong associations with SI. All Axis II parameters differed significantly between the SI and non-SI groups. Patients with SI exhibited higher levels of depression, generalized anxiety, psychological distress, non-specific physical symptoms, pain catastrophizing, and pain-related disability. These findings are consistent with broader evidence linking mood disorders and psychological distress to increased suicide risk [8, 36]. Our results also align with previous studies showing that TMD patients with myofascial pain with referral exhibit significantly higher Axis II psychological factors than those with local myalgia [37, 38]. Similar patterns of elevated Axis II components have been reported in patients diagnosed with headache attributed to TMD [39]. These observations support prior research suggesting that myofascial pain with referral and headache attributed to TMD may be associated with central sensitization, widespread pain, and a potential progression to chronic pain conditions [40, 41, 42, 43, 44].

To the best of our knowledge, this study is the first to apply the DC/TMD criteria to differentiate between local myalgia and myofascial pain with referral in TMD patients reporting SI. This distinction was not possible using the Research Diagnostic Criteria for TMD (RDC/TMD), which includes only a single muscle-related diagnosis (myofascial pain/myofascial pain with limited opening) [45]. Furthermore, the diagnosis of headache attributed to TMD represents an important addition, since this condition is not included among the Axis I diagnoses in the RDC/TMD framework. These findings highlight the need for further research to better understand the role of these conditions in the context of SI among TMD patients.

Another important finding of the present study was the difference in the prevalence of SI across different painful TMD groups. We compared SI prevalence between patients with acute and chronic painful TMD. When chronicity was defined solely by pain duration, SI was significantly more frequent among patients with chronic pain (12%) than among those with acute pain (5.7%). However, when applying the IASP criteria for chronic primary pain, which incorporate psychological distress and pain-related disability in addition to pain duration, the prevalence of SI increased markedly to 20.9%.

Notably, among patients who reported high pain persistence (≥90 pain days in the preceding 6 months) but did not meet the IASP criteria due to low levels of psychological distress and pain-related disability, only two individuals (2.4%) reported SI. This observation suggests that most patients with pain lasting ≥3 months and who report SI also exhibit a substantial psychosocial burden. Accordingly, SI in TMD appears to co-occur with broader psychosocial factors, rather than being related to pain duration alone.

Consistent with this interpretation, the regression analysis revealed that depression, rather than pain persistence, was the factor most strongly associated with SI. These results suggest that the association between chronic TMD and SI may largely reflect the contribution of comorbid depressive symptomatology. Nevertheless, the results of the regression analysis should be interpreted with caution. Although the events-per-variable (EPV) ratio in the model met the commonly suggested threshold (EPV = 12), the small number of SI events in subgroups may have contributed to wide confidence intervals for the pain persistence variables, indicating imprecision and the potential instability of these estimates. Therefore, the absence of a statistically significant association between pain persistence and SI should not be interpreted as evidence of no effect.

In our study, 8.2% of patients with TMD reported SI, a prevalence slightly higher than that reported in most previous studies of TMD populations (1–8.4%) [46, 47]. However, comparisons across studies should be interpreted with caution due to considerable methodological heterogeneity. Differences in international suicide rates related to genetic, ethnic, sociodemographic, cultural, and religious factors [48, 49, 50, 51], variation in SI assessment timeframes (ranging from 1 week to 12 months) [47, 52], and the dichotomous classification of PHQ-9 item 9 may all influence the reported prevalence. Note that this SI assessment using a single dichotomous item may lead to overestimation of the prevalence. Therefore, the reported rates of SI should be interpreted with caution, since they may not fully capture the complexity or severity of suicidal thoughts in this population. Additional variability may arise from differences in diagnostic criteria (*e.g.*, DC/TMD, RDC/TMD, and WHO definitions) and the clinical settings (primary *vs.* tertiary TMD clinics), as well as whether SI is assessed in the overall TMD population or only among patients with chronic TMD. The overall prevalence of SI in our cohort was higher than estimates in the general population (4–6% [2, 3]) but lower than that reported in other chronic pain conditions such as fibromyalgia (29.6%) [13]. However, the prevalence in the IASP-defined chronic painful subgroup (20.9%) was closer to that reported for fibromyalgia, supporting the use of IASP criteria to identify TMD patients with greater chronicity and functional impairment. The intermediate prevalence observed in the total sample likely reflects the heterogeneity of TMD populations, which include patients with non-painful signs, acute localized pain, and chronic pain or comorbid chronic overlapping pain conditions (COPCs) such as fibromyalgia [53, 54]. Consistent with previous studies, fibromyalgia was associated with higher disability and depression than TMD [55, 56], highlighting the psychosocial burden of TMD while indicating that widespread pain conditions generally present with greater severity. These findings further emphasize the importance of avoiding the analysis of TMD patients as a single homogeneous group [57, 58, 59].

In this study, SI was assessed using item 9 of PHQ-9. Although reliance on a single self-reported item may lead to misclassification or false-positive results [60], prioritizing sensitivity may be appropriate in screening contexts to improve the detection of at-risk patients [61]. Although this single item is commonly employed to screen for SI in both clinical and research contexts [62, 63], some studies have questioned the specificity of PHQ-9 item 9, whereas others have shown its association with subsequent suicide attempts and deaths [60, 64]. Additionally, both depression and SI were assessed using PHQ-based instruments at the same time point, raising the possibility of shared method variance and partial construct overlap, which may have inflated the observed association. Therefore, we suggest that within the DC/TMD framework, PHQ-9 item 9 should be considered as an initial screening tool for SI. Assessment of SI in TMD patients should integrate information from the DC/TMD evaluation, including sociodemographic factors, Axis I diagnoses, Axis II psychological measures, and pain persistence, followed by targeted clinical assessment of suicide risk (Fig. 2). In cases of uncertainty, suicide risk severity may be evaluated using other tools, such as the Columbia-Suicide Severity Rating Scale (C-SSRS), a structured and widely validated instrument for assessing the severity of suicidal ideation and behavior across clinical settings [65]. Based on the level of risk identified, patients may require routine monitoring, referral for mental health evaluation, or urgent psychiatric care, within a multidisciplinary biopsychosocial management approach.

**Fig. 2.** Flowchart illustrating a suggested clinical protocol for screening and stratifying SI in patients with TMD. PHQ: Patient Health Questionnaire; DC/TMD: diagnostic criteria for temporomandibular disorders; SI: suicidal ideation; IASP: International Association for the Study of Pain.

## 5. Study limitations

Several limitations should be considered when interpreting the findings of this study:

1. The retrospective cross-sectional design prevents establishing temporality between depressive symptoms, chronic TMD pain, and SI. Further prospective studies are warranted to clarify the temporal sequence of these relationships and to validate the utility of psychosocial screening recommendations for identifying TMD patients at risk of SI.

2. The study population was derived from a single tertiary academic orofacial pain clinic, which may limit the generalizability of the findings and introduce referral and selection bias. Patients treated in such specialized settings may represent more severe or complex TMD cases and may have a higher psychosocial burden. However, although this bias may influence prevalence estimates, it is less likely to substantially affect the observed associations. Future multicenter studies including diverse clinical settings are recommended to reduce potential referral bias and improve the generalizability of the findings.

3. SI was assessed using only screening item 9 of PHQ-9, and a structured psychiatric suicide risk assessment was not systematically conducted for research purposes. This may introduce misclassification bias and limits the ability to evaluate SI more comprehensively, for example, differentiating between passive and active SI or capturing information on suicide plans or prior suicide attempts. In addition, due to the relatively small number of participants, further subdivision into frequency-based categories resulted in very small subgroups, consequently limiting the statistical power and increasing the likelihood of unstable estimates. Therefore, SI was analyzed as a dichotomous variable. Future studies with larger samples and more comprehensive suicide risk assessments could provide a more detailed characterization of SI in TMD populations and enable analysis of SI severity using ordinal or continuous measures.

4. The self-report nature of this study introduces potential reporting bias; moreover, varied missing responses across different variables may contribute to selection and non-response biases. Nevertheless, sensitivity analyses restricted to participants with complete data did not alter the direction or statistical significance of the main associations. Future studies should complement self-report measures with objective clinical assessments or structured interviews to reduce these potential biases.

5. The operationalization of IASP chronic primary pain in this study was based on an adaptation of the DC/TMD Axis II assessment tools, rather than a validated protocol. Accordingly, its sensitivity and specificity are unknown, and the potential for misclassification bias should be considered when interpreting the findings. Further research is required to validate its use in defining chronic primary TMD pain while integrating the psychosocial factors taken from DC/TMD.

## 6. Conclusions

The present study identified an increased prevalence of SI, particularly among patients with chronic primary TMD, as defined by the IASP. To the best of our knowledge, this is the first study to apply the DC/TMD protocol to evaluate Axis I diagnoses in patients reporting SI, while also integrating psychosocial parameters into the IASP framework for chronic primary TMD pain. Further research is needed to validate the use of the DC/TMD Axis II protocol in operationalizing IASP criteria for chronic primary pain, as proposed in this study.

Although this study is based on a tertiary care population and may therefore have limited generalizability, the findings suggest that considering TMD as a single diagnostic entity may mask higher rates of SI within specific subgroups. The increased prevalence of SI appears to be driven primarily by psychosocial factors (Axis II), rather than by pain duration alone, underscoring the importance of incorporating psychosocial dimensions into the definition of chronic primary painful TMD.

Although PHQ-9 Item 9 may serve as an initial screening tool for SI, positive responses should be followed by a comprehensive assessment and a formal mental health evaluation. Routine screening for SI in TMD populations, particularly among those with chronic primary TMD, may facilitate the identification of high-risk individuals and enable timely referrals for appropriate evaluation and intervention.
